# What is science without replication?

**DOI:** 10.1007/s40037-016-0307-z

**Published:** 2016-10-26

**Authors:** Jimmie Leppink, Patricia Pérez-Fuster

**Affiliations:** 1 School of Health Professions Education, Maastricht University, Maastricht, The Netherlands; 2 Faculty of Psychology, University of Valencia, Valencia, Spain

The Latin adagio *unus testis nullus testis* is an important principle that equally applies to criminal law as to science: we should not base our conclusions on a single piece of evidence [1]. Of course, criminal law and science are very different practices. After all, while a criminal case typically revolves around the evaluation of evidence in favour of and against competing hypotheses about what occurred in a particular case with specific actors, the goal of science is to establish generally applicable laws and principles. Yet, what unites the two practices is that both are about establishing a *chain of evidence*: pieces of evidence have to be anchored as narratives into a story line that increases the plausibility of some hypothesis relative to competing hypotheses. Just like a DNA match from a cigarette found at a crime scene cannot be sufficient to conclude on the guilt of the suspect in the absence of contextual information on how the cigarette got there (e. g. eyewitness testimonies), the meaning of findings from a scientific study cannot be established without considering context, theory and relevant previous research. In other words, science too is about storytelling [2].

In their article *Science: The slow march of accumulating evidence* [3], Picho, Maggio and Artino discuss a very powerful if not the most powerful tool for establishing chains of evidence and solid stories in a scientific research field: replication. We fully support the authors’ plea to treat replication as essential to the accumulation of knowledge in the field of medical education and would like to take it even one step further by arguing that the potential of replication is even bigger than discussed in the vast body of literature on replication in education and psychology thus far.

## Why the potential of replication is even bigger than discussed thus far

Picho and colleagues distinguish between direct and conceptual replication. While the former comes down to repeating a study as closely as possible, the latter is about attempts to test the theory that underlies particular findings. The authors provide excellent arguments for why especially conceptual replication can help improve the quality of research in a field. Take for instance the so-called *expertise reversal effect* [4]: instructional support that is beneficial for novice learners loses its effectiveness or even becomes detrimental as learners become more proficient. This effect has been replicated for different types of learners in different domains and therefore has clear implications for educational practice and research in these domains.

However, we would like to argue that both direct and conceptual replication have a use for at least one common reason: different studies are carried out with different participants and therefore always yield somewhat different results. In sampling theory, this is also referred to as *sampling error*. Under the assumption of random sampling, which underlies the frequently reported *p*-values and many other statistics, findings from individual studies and hence differences between studies are to some extent always due to chance. This sample-to-sample fluctuation is especially large when dealing with small samples, something that is quite common in medical education research (e. g. only ten residents or twenty students). In small samples, estimates of parameters of interest (e. g. means, correlations and regression coefficients) can vary considerably from sample to sample [5] and both *false positive* and *false negative* rates tend to be elevated [6]. Both direct and conceptual replication can in such a context help obtain more stable estimates (statistics) and reduce both types of erroneous hypothesis testing decisions [7].

Although replication, and direct replication in particular [3], is commonly associated with repeating experimental procedures, it is also very useful in the context of instrument development. The latter is often forgotten and that is unfortunate because the stability of factors in a questionnaire, test or assessment tool is established in a series of studies rather than in a single study. That is, if we have a questionnaire of say ten items that are expected to form three factors, the same sets of items should pop up in psychometric analysis in different samples [8]. If we fail to replicate this factor structure in subsequent samples, we cannot assume stable factors let alone that our factors consistently measure the constructs we are interested in. Moreover, the use of psychometric instruments introduces a source of error next to sampling error, namely *measurement error*: fluctuation of scores across administrations of a given instrument due to imperfect reliability of that instrument. This type of error adds to sample-to-sample variation and hence even more underlines the need for replication studies.

Where replications can help researchers obtain more stable statistics and reduce false positive and false negative rates when dealing with quantitative data, in the context of qualitative data replication studies can help for instance to assess whether indeed saturation was reached in an initial study. After all, if researchers in a qualitative study decide at some point that further data collection is not needed because saturation has been achieved, a replication of the study with a very similar group of participants should yield very similar results.

## What steps must be taken to enable and facilitate replication

From the previous, it becomes clear that whether we are dealing with quantitative, qualitative or mixed-methods data, it is of paramount importance that all choices and decisions made throughout the study – prior to and after data collection – be made explicit [9–11]. Although this is of course typically much more difficult for qualitative than for quantitative data, the findings from the Reproducibility Project [12] make very clear that establishing generally applicable laws and principles cannot reasonably be achieved without replication. Whether we are interested in measuring learning outcomes from training of medical specialists or in experiences of anxiety in a particular patient population, the stakes are high and thus studies need to be replicated to maintain and improve the quality of our research [9–11] and the implications for practice and further study that come forth from that research.

Finally, with regard to quantitative data, we need to study the test-retest reliability of our questionnaires, tests and other psychometric instruments more than we have done thus far [13]. Cronbach’s alpha and other statistics used as indicators of the reliability of measurements at a single point in time tell us close to nothing about the extent to which the repeated use of our instruments would result in similar outcomes. The extent to which repeated use of our instruments results in similar outcomes is indicated by a good test-retest reliability and is a necessary condition for meaningful replication research, even if these replication studies do not include the same group of participants. An instrument supposed to measure medical students’ motivation to learn about medicine resulting in totally unrelated scores for the same students in two consecutive weeks would not have a good test-retest reliability and should thus not be used in studies that intend to measure this construct.

## To conclude

If the goal of science is to establish generally applicable laws and principles, replication is a *conditio sine qua non*: without replication, we cannot establish whether findings obtained in a given study are artefacts or actually reflect laws and principles that have a certain applicability outside the given study. The plea by Picho and colleagues to put replication more at the forefront is a very welcome and timely contribution to the discussion on an issue of fundamental importance to the field of medical education. We fully support their arguments in favour of replication, applaud efforts by journals such as this one to give more opportunities to researchers for replication research, and hope that more researchers will use this space. With regard to the latter, making all choices and decisions made throughout the study explicit and an increased focus on test-retest reliability of instruments used will provide the necessary guidance.
